# Electrodeposition of amorphous molybdenum sulfide thin film for electrochemical hydrogen evolution reaction

**DOI:** 10.1186/s13065-019-0600-0

**Published:** 2019-07-10

**Authors:** Lina Zhang, Liangliu Wu, Jing Li, Jinglei Lei

**Affiliations:** School of Chemistry and Chemical Engineering, Chongqing University, Chongqing, 400044 People’s Republic of China

**Keywords:** Thiomolybdates solution, Amorphous molybdenum sulfide, Buffer solution, Electrodeposition, HER

## Abstract

Amorphous molybdenum sulfide (MoS_x_) is a highly active noble-metal-free electrocatalysts for the hydrogen evolution reaction (HER). The MoS_x_ was prepared by electrochemical deposition at room temperature. Low-cost precursors of Mo and S were adopted to synthesize thiomolybdates solution as the electrolyte. It replaces the expensive (NH)_2_MoS_4_ and avoid the poison gas (H_2_S) to generate or employ. The (MoO_2_S_2_)^2−^, (MoOS_3_)^2−^ and (MoS_4_)^2−^ ions were determined by UV–VIS spectroscopy. The electrodeposition of MoS_x_ was confirmed with XRD, XPS and SEM. The electrocatalyst activity was measured by polarization curve. The electrolyte contained (MoO_2_S_2_)^2−^ ion and (MoOS_3_)^2−^ ion electrodeposit the MoS_x_ thin film displays a relatively high activity for HER with low overpotential of 211 mV at a current density of 10 mA cm^−2^, a relatively high current density of 21.03 mA cm^−2^ at *η *= 250 mV, a small Tafel slope of 55 mV dec^−1^. The added sodium dodecyl sulfate (SDS) can efficient improve the stability of the MoS_x_ film catalyst.

**Electronic supplementary material:**

The online version of this article (10.1186/s13065-019-0600-0) contains supplementary material, which is available to authorized users.

## Introduction

Hydrogen is a cleaner and sustainable energy, and it is one of the promising alternative energy carriers [1, 2]. Electrochemical water splitting is attractive methods for hydrogen evolution [3–5]. An important problem for this method is seeking highly catalytic active electrocatalysts for hydrogen evolution reaction. In this regard, various efficient electrocatalysts materials, including Pt and other noble metals were investigated. However, high cost of Pt or other noble metals impede their widespread application [6, 7].

The employment of catalysts should have greatly highly catalytic active, low-cost, and earth-abundant non-noble metal. Recently, molybdenum sulfide is found to be an active HER catalyst, and it is useful for acidic HER condition [8–20]. While amorphous molybdenum shows highly catalytic activity at the unsaturated sulfur atoms present over the entire surface [11, 13, 21–25]. In the previous research, the most promising method of preparing the amorphous materials is cathodic reduction of an aqueous solution of ammonium tetrathiomolybdate ((NH)_2_MoS_4_). Some researchers used the commodity ((NH)_2_MoS_4_) [13, 21, 25–27], however, the commodity ((NH)_2_MoS_4_) is highly expensive, therefore, some researchers synthesize the ((NH)_2_MoS_4_) solution [28–30]. The methods for preparing of ammonium tetrathiomolybdate ((NH_4_)_2_(MoS_4_)) species are almost identical to Krüss [29], and the methods was improved by John W. McDonald’s group [30] for the preparation of (NH_4_)_2_(MoO_2_S_2_), (NH_4_)_2_(MoOS_3_) and (NH_4_)_2_(MoS_4_). The synthesis involves the exhaustive treatment by H_2_S gas of molybdate solution in concentrated NH_4_OH. This method can easy to obtain the (NH_4_)_2_(MoS_4_), however, a steady stream of H_2_S was employed. Ponomarev et al. [28] prepared the tetrathiomolybdate solution utilized a chemical reaction route. To a mixture solution of 5 mmol L^−1^ Na_2_MoO_4_ and excess Na_2_S was added hydrochloric acid with stirring until a pH of 8.0 was attained. During this process, large amount of H_2_S gas was generated.

In this work, we further improved the approaches of synthesis of thiomolybdates solution. (NH_4_)_6_Mo_7_O_24_·4H_2_O and Na_2_S·9H_2_O were employed as the precursors of Mo and S, respectively. The ammonium chloride buffer solution (pH = 8) replaced the hydrochloric acid to make the pH of the solution to 8. This method does not produce a large amount of H_2_S gas due to excessive local acid concentration. And it is very simple, the process is easy to control and is mild. Additionally, the precursor materials are economic, especially, the prepared thiomolybdates solution has great stability. The synthesized thiomolybdates solution as the electrolyte, employ the electrochemical deposition of amorphous molybdenum sulfide thin film for electrochemical hydrogen evolution. The HER performance measurement result suggests the catalyst displayed high catalytic activity for hydrogen evolution reaction. Add a bit of surfactant into the electrolyte, the stability of the MoS_x_ film has effectively improved.

## Materials and methods

### Materials

Hexaammonium heptamolybdate tetrahydrate ((NH_4_)_6_Mo_7_O_24_·4H_2_O, ≥ 99.0%) was used as the Mo precursor. Sodium sulfide nonahydrate (Na_2_S·9H_2_O, ≥ 98.0%) was used as the S precursor. Ammonium chloride (NH_4_Cl, ≥ 99.5%), ammonia solution (NH_3_, 25–28%), sulfuric acid (H_2_SO_4_, 95–98%), hydrochloric acid (HCl, 36.0–38.0%), acetone (CH_3_COCH_3_, ≥ 99.5%), sodium dodecyl sulfate (C_12_H_25_NaO_4_S, ≥ 85.0%). All reagents were purchased and used as received.

UV–VIS spectrophotometer (TU-1810,Beijing). Scanning electron microscopy (SEM) combined with energy dispersive X-ray spectroscopic (EDS) images were taken with a TESCAN VEGA II LMU instrument. The phase compositions of the samples were identified using an X-ray diffractometer (XRD, X’pert PRO, PANalytical B.V., Holland) using Cu Kα radiation (0.15418 nm). The electrodeposition and electrochemical measurements were carried out at room temperature in a three-electrode glass cell connected to an electrochemical workstation (CHI440A, chenghua, Shanghai). The surface chemical composition was analyzed by X-ray photoelectron spectroscopy (XPS, Thermoelectron ESCALAB 250, USA).

### Syntheses of thiomolybdates solution

(NH_4_)_6_Mo_7_O_24_·4H_2_O (3.58 g) was dissolved in 200 mL ammonium chloride buffer solution (pH = 8). In a second container, 21.65 g of Na_2_S·9H_2_O was added to 300 mL of ammonium chloride buffer solution (pH = 8). These two solutions were mixed and transferred to a 500 mL beaker. Put the mixed solution beaker to the ~  90 °C water bath for 2 h. After that, the black and red solution was transferred to a 500 mL flask. Once the solution is cooling down to the room temperature, then the deionized water is used to add the solution to the scale.

### Catalyst synthesis

The substrate used titanium ingot (11.28 mm diameter, 3.5 mm thick, purity 99.99%). Prior to the electrodeposition, the Ti substrate was carefully cleaned with mechanical polishing, acetone and HCl solution (9 wt%) in an ultrasound bath each for 5 min, successively. And then it was washed with deionized water after each step. Polytetrafluoroethylene (PTFE) electrode sets with working area of 1 cm^2^. MoS_x_ was deposited on Ti substrate by electrodeposition in a three-electrode setup. The PTFE electrode sets with treated Ti substrate as the working electrode, saturated calomel electrode (SCE) as the reference electrode, and a graphite board as the counter electrode. The synthesized thiomolybdates solution as the electrolyte. The electrodeposition adopted the method of chronopotentiometry (CP).

### Spectroscopic characterization

The thiomolybdates determination were conducted using the UV–VIS spectrophotometer of ref. 30. Take 0.1 mL thiomolybdates solution and dilute to 100 mL for spectral detecting. The range of wavelength is from 190 to 600 nm. The scan rate is 0.5 nm s^−1^.

### Electrochemical measurements

Electrochemical measurements were carried out with a three-electrode configuration in which saturated calomel electrode as the reference electrode, a graphite board as the counter electrode. Linear sweep voltammetry (LSV) with a 5 mV s^−1^ scan rate was performed in 0.5 M H_2_SO_4_ electrolyte, which was purged with N_2_ gas for at least 30 min prior to the LSV measurements in order to remove any dissolved O_2_. LSV curves were measured fifth for each sample to verification of the system’s chemical stability. The scan range from 0.00 to − 0.55 V vs. SCE (not *iR* corrected). After the LSV measurements, the solution was stirred. The reference electrode was calibrated for the reversible hydrogen potential using platinum wire was working and counter electrodes in the electrolyte solution saturated with H_2_. In 0.5 M H_2_SO_4_, the potential was converted to the reversible hydrogen potential (RHE) reference electrode by E (vs. RHE) = E (vs. SCE) + 0.26 V. The resistance (R) was tested by EIS. EIS measurements were carried out in the frequency range of 0.1 Hz to 10^5^ Hz under a hydrogen evolution voltage, which corresponds to the potential at 10 mA cm^−2^.

Electrochemical stability is an important parameter for viability of a HER catalyst. To investigate HER stability under electrocatalytic operation in the acidic environment, long-term potential cycling stability of the MoS_x_ film was assessed by taking continuous cyclic voltammograms (CV) between 0.0 and − 0.55 V vs. saturated calomel electrode (not *iR* corrected) at 100 mV s^−1^.

## Results and discussion

### Electrolyte

**Fig. 1:**
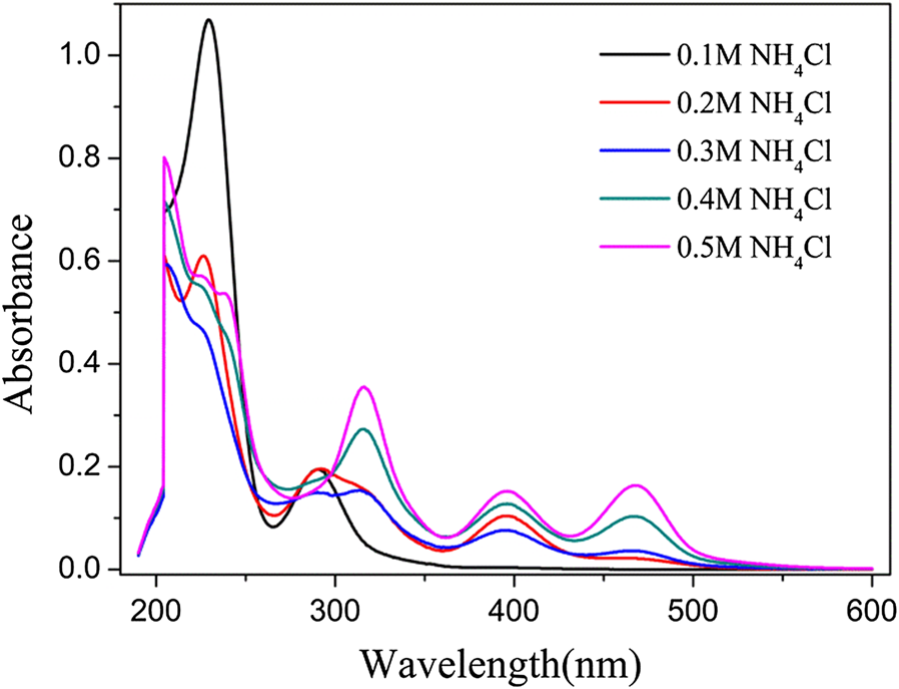
Electronic spectra of thiomolybdates solutions with different concentration of ammonium chloride in the ammonium chloride buffer solution

Thiomolybdates solutions were synthesized in the buffer solutions containing different concentrations of ammonium chloride. The ammonium chloride concentration is from 0.1 to 0.5 M. Different ammonium chloride concentration results in the different color of the thiomolybdates solutions. The thiomolybdates solutions color was changed from light yellow to dark red, along with the increasing of the ammonium chloride concentration. The different color of the thiomolybdates solutions attribute to the different thiomolybdates species. The various thiomolybdates can be determined by UV–VIS Spectroscopy [30]. The actual UV–VIS Spectra of the thiomolybdates solutions are shown in Fig. 1. Peak position and molar absorptivities are provided in Table 1.

**Table 1:** Spectral data for thiomolybdates solutions

Concentration of ammonium chloride	UV–VIS^a^
0.1 M	229.5(1.069), 290.5(0.195)
0.2 M	226.5(0.610), 292.0(0.196), 395.5(0.104)
0.3 M	290.5(0.150), 313.0(0.154), 396.0(0.076), 466.00(0.036)
0.4 M	315.5(0.273), 396.0(0.128), 467.0(0.103)
0.5 M	316.5(0.355), 396.0(0.152), 468.0(0.163)

^a^Peak positions in nm with molar absorbance in parentheses

By comparing the results from the previously reports [30], it can be concluded that to adjust the ammonium chloride concentration of the ammonium chloride buffer solution can syntheses the various thiomolybdates solutions. With the concentration of ammonium chloride increases, the thio-degree rises up. In the 0.2 M NH_4_Cl buffer solution, the molar absorptivities for the peaks at 292.0 and 395.5 nm, the result clear support for the (MoO_2_S_2_)^2−^ ion was synthesized. In the 0.3 M NH_4_Cl buffer solution, the peak at 466 nm started to appear, this result supports for the (MoOS_3_)^2−^ ion initial synthesis. In the solutions with ammonium chloride concentration of 0.4 M and 0.5 M, the intensity of the peak at 467.0 nm is becoming stronger, and the intensity of the peaks at 396.0 and 467.0 nm was very close. From the previously reports [30], the purity (MoS_4_)^2−^ ion exhibits a very strong absorption at 467 nm but non at 395 nm. In Fig. 1, according to the spectra of the 0.4 M NH_4_Cl and the 0.5 M NH_4_Cl buffer solution, the peaks at 396.0 and 467.0 nm are simultaneous occurrence. From these results it is clear that the solution contains both of the (MoOS_3_)^2−^ ion and (MoS_4_)^2−^ ion, and the content of (MoS_4_)^2−^ in the 0.5 M NH_4_Cl buffer solution is more than in the 0.4 M NH_4_Cl buffer solution. The ammonium chloride concentration determines the buffer capacity of buffer solution. The results suggest both of the 0.4 M and 0.5 M NH_4_Cl buffer solution could synthesize the solution with the (MoOS_3_)^2−^ ion and (MoS_4_)^2−^ ion. And the two ions could to produce the molybdenum sulfide thin film under electrochemical deposition. We required the synthesized thiomolybdates solution as the electrolyte to electrodeposit of molybdenum sulfide thin film, and the molybdenum sulfide thin film could with relatively high HER performance.

### Characterization of MoS_x_

In the previous studies [13, 21, 25–27], they always employed the purity tetrathiomolybdate to prepare the MoS_2_ or MoS_3_. In this work, we applied the synthesized thiomolybdates solution as the electrolyte to electrodeposit of molybdenum sulfide thin film for electrochemical hydrogen evolution, and XRD (Additional file 1: Figure S1) analysis identified as amorphous molybdenum sulfides.

**Fig. 2:**
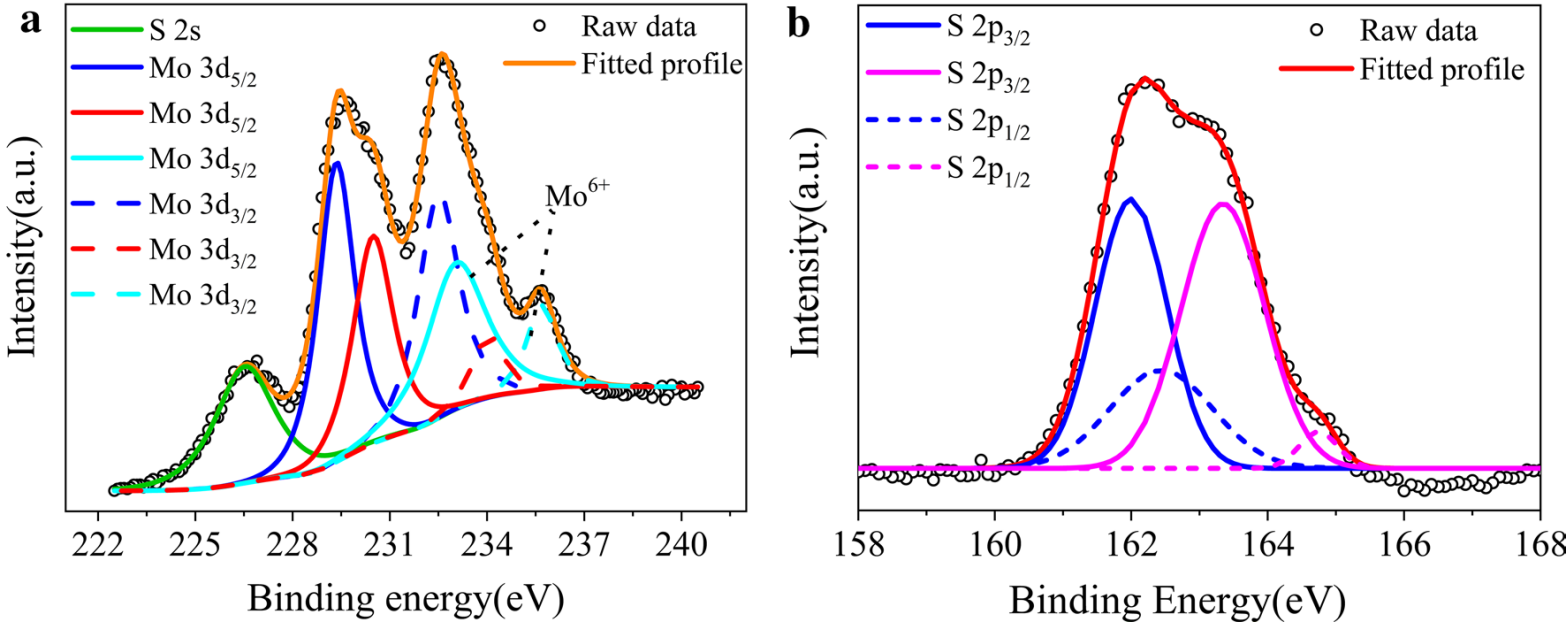
XPS spectra for MoS_x_ film grown by chronopotentiometry negative electrodeposition at 2 mA cm^−2^. **a** Mo 3d and S 2 s region; experimental data (circle line), fitting envelope (orange line), Mo^4+^ (blue line), Mo^5+^ (red line), Mo^6+^ (light blue line), S^2−^ 2s (green line), **b** S 2p region; experimental data (circle line), fitting envelope (orange line), S_2_^2−^ (purple line), S^2−^ (blue line)

Figure 2 displays the detailed XPS scans for the Mo and S binding energies for the molybdenum sulfide thin film. The XPS spectra of molybdenum sulfide thin film are similar to those of known MoS_x_ samples [13, 22]. The molybdenum sulfide thin film exhibits two characteristic peaks at 229.4 and 232.5 eV, attributed to the Mo 3d_5/2_ and 3d_3/2_ binding energies for Mo^4+^ [11, 13, 22]. The observation of Mo 3d_5/2_ and 3d_3/2_ binding energies at 230.5 and 234.1 eV suggests the presence of Mo^5+^ ions [11, 13, 22]. The peaks, corresponding to the Mo 3d_5/2_ and 3d_3/2_ orbital of Mo^6+^ are observed at 233.1 and 235.7 eV. Meanwhile, the S 2p_1/2_ and 2p_3/2_ energies at 162.0 and 162.4 eV demonstrate the existence of bridging S^2−^. And the S 2p_1/2_ and 2p_3/2_ energies at 163.3 and 164.7 eV indicate the existence of bridging S_2_^2−^ or S^2−^. The binding energies of Mo and S, proving that the structure is amorphous molybdenum sulfides, labeled as MoS_x_ [22, 31].

### Electrodeposition MoS_x_

**Fig. 3:**
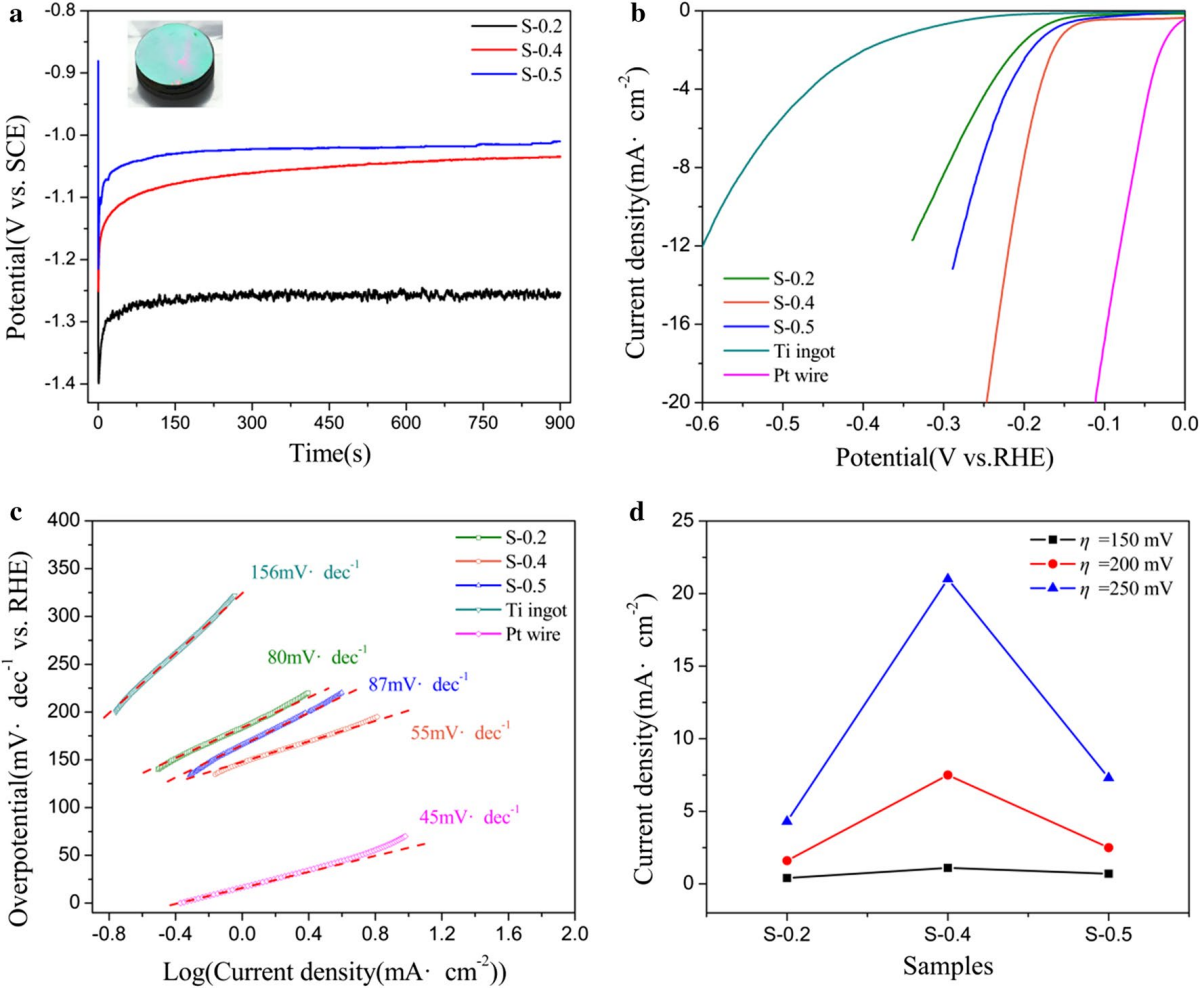
**a** Chronopotentiometry during the deposition of molybdenum sulfide films, the samples named as S-0.2, S-0.4 and S-0.5 corresponding to the ammonium chloride concentration with 0.2 M, 0.4 M and 0.5 M, respectively. Inset: digital photo of an amorphous molybdenum sulfide film on Ti ingot. **b** Polarization curves for HER on bare Ti ingot and deposition on the Ti ingot of MoS_x_ films of S-0.2, S-0.4 and S-0.5 and a high-pure Pt wire, scan rate = 5 mV s^−1^. **c** Tafel plot for the various catalysts derived from **b**. **d** Current densities curves at the overpotential of 150 mV, 200 mV and 250 mV, respectively

The electrodeposition method for amorphous molybdenum sulfide thin film was CP. The deposition current density was 2 mA cm^−2^, the deposition temperature was 20.0 °C, the deposition time was 900 s, and accompanied with stirring during the deposition process. The electrolyte used the synthesized thiomolybdates solutions with 0.2 M, 0.4 M and 0.5 M ammonium chloride, respectively. The samples named as S-0.2, S-0.4 and S-0.5 corresponding to the ammonium chloride concentration. The deposition curves (potential–time) are shown in Fig. 3a, and color film formed on the electrode (Inset in Fig. 3a).

### HER activities

The HER catalytic activity of these molybdenum sulfide films as the catalyst was measured employing the standard three-electrode electrochemical configuration in 0.5 M H_2_SO_4_ electrolyte-aerated with Ar, as described in “Materials and methods”. The polarization curves (not *iR* corrected) showing the normalized current density versus voltage (*j* versus V) for the S-0.2, S-0.4 and S-0.5 films along with Pt wire and Ti ingot samples, for comparison, are illustrated in Fig. 3b. As expected, Pt wire catalyst exhibits excellent HER performance, and their HER performances are summarized in Table 2. In contrast, S-0.2, S-0.4 and S-0.5 films produces *j* of 10 mA cm^−2^ at *η* of 319 mV, 211 mV and 270 mV, respectively. Further insight into the catalytic activity of MoS_x_ samples were obtained by extracting the slopes from the Tafel plots shown in Fig. 3c. The corresponding Tafel slopes of the MoS_x_ films are in the range of 55 to 87 mV dec^−1^. The lowest Tafel slope of ~  55 mV per decade was attained for the sample of S-0.4. This indicates the Volmer reaction is taking place, a process to convert protons into sorbed hydrogen atoms on the MoSx film surface, and this process becomes the rate-determining step in the HER mechanism [5, 32, 33]. Figure 3d exhibits the ammonium chloride concentration dependent current densities at *η *= 150, 200 and 250 mV. The current densities at the optimal ammonium chloride concentration are 1.12, 7.50 and 21.03 mA cm^−2^ at *η *= 150, 200 and 250 mV, respectively. The optimal ammonium chloride concentration is 0.4 M. The sample of S-0.4 film displayed relative high catalytic activity for hydrogen evolution reaction, the overpotential is lower than many other reported acid-stable and earth-abundant HER electrocatalysts, including amorphous MoS_3_ (~ 270 mV at 10 mA cm^−2^) [11], amorphous MoS_x_ film (~ 150 mV at 0.4 mA cm^−2^) [21], amorphous molybdenum sulfide (~ 200 mV at 10 mA cm^−2^) [23], electrodeposited MoS_2_ (~ 440 mV at 10 mA cm^−2^) [24] and double-gyroid mesoporous MoS_2_ films (~ 235 mV at 10 mA cm^−2^) [34] (More details of HER parameters of MoS_x_ and other literature values is listed in Table 3).

**Table 2:** Comparison of catalytic performance of different HER electrocatalysts in 0.5 M H_2_SO_4_

Catalyst	Exchange current density (μA cm^−2^)	*j* (mA cm^−2^) *η *= 150 mV	*j* (mA cm^−2^) *η *= 200 mV	*j* (mA cm^−2^) *η *= 250 mV	Overpotential *η* (mV vs. RHE) *j *= 10 mA cm^−2^	Tafel slop (mV dec^−1^)
Pt wire	429.89	32.290	56.660	–	72	45
S-0.2	5.088	0.387	1.560	4.338	319	80
S-0.4	1.89	1.117	7.501	21.030	211	55
S-0.5	12.37	0.671	2.508	7.268	270	87

**Table 3:** HER parameters of MoS_x_ and other literature values

Catalysts	Exchange current density (μA cm^−2^)	*j* (mA cm^−2^)	Overpotential *η* (mV vs. RHE) *j *= 10 mA cm^−2^	Tafel slop (mV dec^−1^)
Amorphous MoS_x_ film (this work)	1.89	21.030 *η *= 250 mV	211	55
Amorphous MoS_3_ [11]	–	1.2 ~ 1.0 *η *= 200 mV	~ 270	41 ~ 63
Amorphous MoS_3_ [13]	–	–	160	40
Amorphous MoS_3_-AE [25]	–	–	~ 170 mV *j *= 20 mA cm^−2^	–
Amorphous MoS_3_-CV film [21]	0.13	0.4 *η *= 150 mV	200 *j *= 14 mA cm^−2^	40
Amorphous molybdenum sulfide [23]	–	–	~ 200	53 ~ 65
Electrodeposited MoS_2_ [24]	–	0.34 *η *= 200 mV	~ 440	106
MoS_2_ sheet [8]	200	–	104	59
Double-gyroid MoS_2_ films [34]	0.7	–	~ 235	50
MoO_3_-MoS_2_ nanowires [35]	–	20 (*iR* corrected) *η *= 270 mV	320	50 ~ 60
MoS_2.7_@NPG [36]	–	–	210	41

**Fig. 4:**
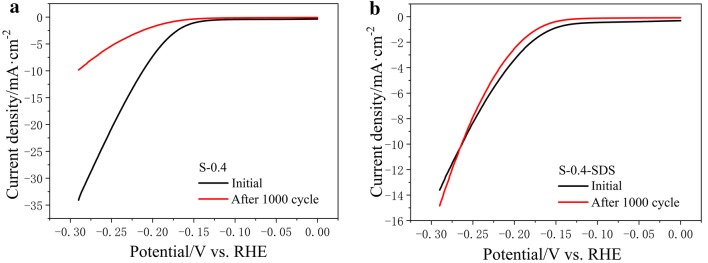
**a** The polarization curves of S-0.4 before and after CV for 1000 cycles in 0.5 M H_2_SO_4_ solution. **b** The polarization curves of S-0.4-SDS before and after CV for 1000 cycles in 0.5 M H_2_SO_4_ solution

**Fig. 5:**
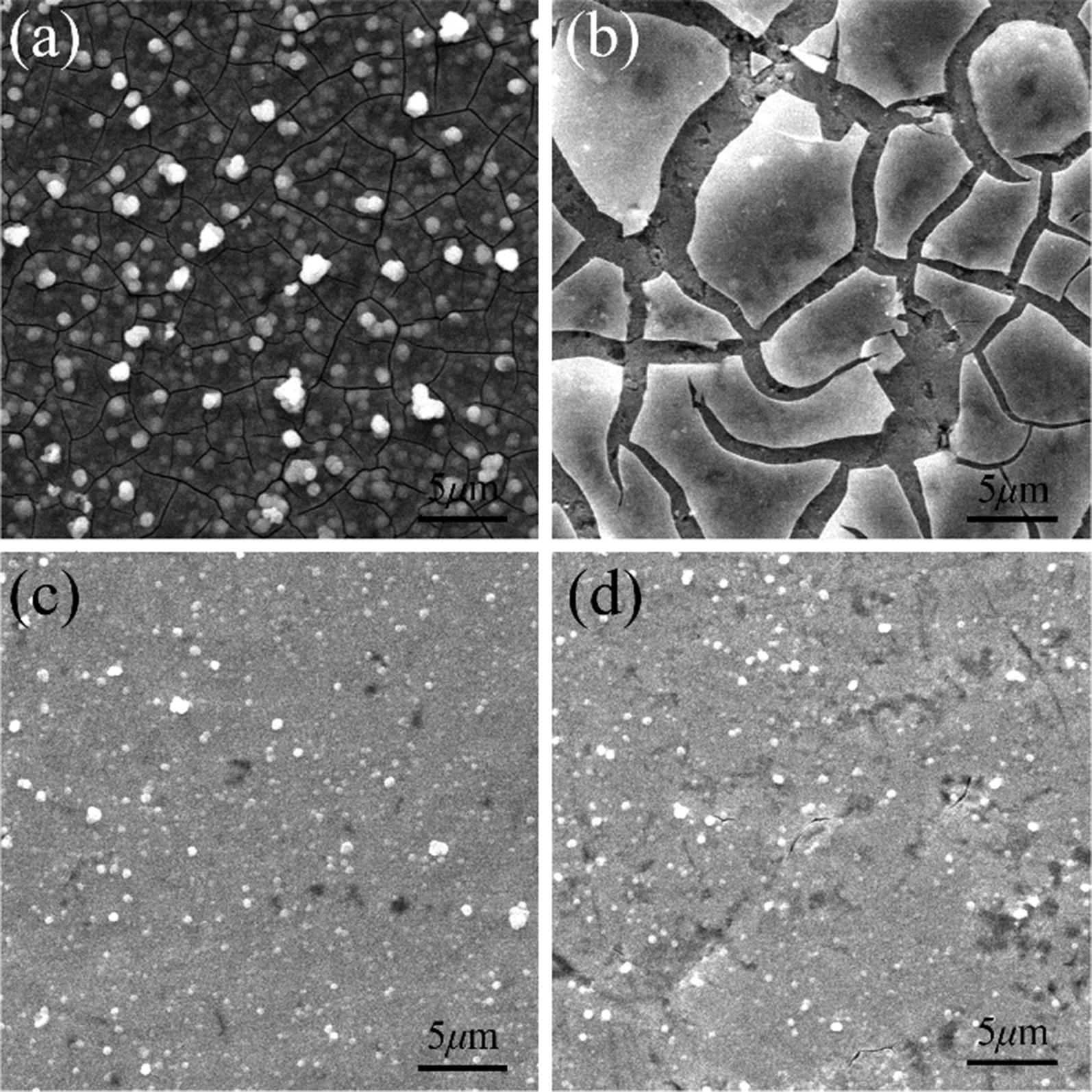
SEM images of amorphous MoSx films. Panels a and b are the SEM images for S-0.4 **a** before and **b** after CV for 1000 cycles. Panels c and d are the SEM images for S-0.4-SDS **c** before and **d** after CV for 1000 cycles

Another important aspect utilized to evaluate the performance of an electrocatalyst is the long-term operating stability. Continuous cyclic voltammetry (CV) in the cathodic potential window at a scan rate of 100 mV s^−1^ was performed on the films over 1000 cycles to investigate their long-term stability. Cathodic polarization curves were collected after 1000 cycles testing (Fig. 4) to investigation the current–density degradation compared with the initial polarization curve. In Fig. 4a, the cathodic polarization curves were corresponding to the sample of S-0.4. It is observed that the current density (without *i*R correction at overpotential of 250 mV) degradation from 20.72 mA cm^−2^ to 5.34 mA cm^−2^ (ca. 26% retention) after 1000 cycles. This suggests that the sample of S-0.4 was not stable enough. To improve the stable of the sample, a little surfactant was added into the thiomolybdates solution electrolyte. The purpose is to reduce the surface tension of the electrode, and allows the deposited sample to have better adhesion. Among a wide variety of surfactants, sodium dodecyl sulfate (SDS) was accepted. The concentration of SDS in the thiomolybdates solution was 5 mM. With the same condition of S-0.4, the sample added SDS labeled as S-0.4-SDS. And the cathodic polarization curves were collected of the sample S-0.4-SDS shown in Fig. 4b. From the curves, the current density (without *i*R correction at overpotential of 250 mV) degradation from 8.31 to 7.87 mA cm^−2^ (ca. 95% retention) after 1000 cycles. This demonstrates that the S-0.4-SDS films are stable throughout long-term repeated cycling in acidic electrolyte. The HER catalytic activity of the sample of S-0.4-SDS was studied by polarization measurements. The current densities are 0.86, 3.37 and 8.31 mA cm^−2^ at *η *= 150, 200 and 250 mV, respectively. The Tafel slop is about 80 mV dec^−1^. Although the Tafel slop was higher, the stable of the catalytic was much more improved. Furthermore, SEM images performed on the two samples (Fig. 5) both of their before and after cycles. The SEM images confirms that the surface morphology of S-0.4-SDS (Fig. 5c) and was not changed after 1000 cycles (Fig. 5d). In addition, the energy-dispersion X-ray spectroscopy (EDS) images (Additional file 1: Figure S3h, i, k, l) showed homogeneous distribution of Mo and S elements. But the surface morphology of S-0.4 (Fig. 5a) was appeared many deep cracks after 1000 cycles (Fig. 5b) with corresponding EDS mapping (Additional file 1: Figure S3b, c, e, f) uniform distribution for Mo and S elements. The SDS is one of the surface active agent. Adding appropriate surfactant can decrease the surface tension of the MoSx film, increase the dispersion and minish effectively particle size of MoSx film, thereby improve effectively the stability of the MoSx film.

Meanwhile, electrochemical impedance spectroscopy (EIS) was employed to evaluate the conductivity of the catalysts (Additional file 1: Figure S2). The Nyquist plots were fitted using an equivalent circuit containing a resistor (Rs) in series with two parallel units, a charge-transfer resistance (Rct) and a constant phase element (CPE1), where Rs represents the solution resistance. The Rs values of S-0.4, S-0.4-SDS, and Ti ingot are 1.546, 1.477 and 1.146 Ω, respectively. The observed semicircle is mainly ascribed to the Rct of H^+^ reduction at the electrode–electrolyte interface. The Rct values of S-0.4, S-0.4-SDS, and Ti ingot are estimated as 1.762, 1.941 and 47.600 Ω from the diameter of the semicircles, respectively. A smaller Rct value represents a faster reaction rate in the catalytic process. The EIS results could further explain the S-0.4 and S-0.4-SDS presented a charge-transfer resistance (Rct) obviously lower than those of Ti ingot. The result is consistent with the polarization curve.

## Conclusions

In conclusion, we have developed a low-cost, environmentally friendly and a simple synthetic strategy to synthesis of thiomolybdates solution as the electrolyte to electrodeposit of amorphous molybdenum sulfide thin film for the HER. Our results provide evidence for electrodeposit of amorphous molybdenum sulfide thin film not only can used the electrolyte consists purity (MoS_4_)^2−^ ion but also the (MoO_2_S_2_)^2−^ ion and the (MoOS_3_)^2−^ ion consists in the electrolyte can electrodeposit the amorphous molybdenum sulfide thin film. The electrolyte contained (MoO_2_S_2_)^2−^ ion and (MoOS_3_)^2−^ ion electrodeposit the MoS_x_ thin film displays a relatively high activity for HER with low overpotential of 211 mV at a current density of 10 mA cm^−2^, a relatively high current density of 21.03 mA cm^−2^ at *η *= 250 mV, a small Tafel slope of 55 mV dec^−1^. When the SDS is added into the electrolyte, the stability of the MoS_x_ film has effectively improved, even though the catalytic activity for hydrogen evolution reaction has reduced. Therefore, this work essentially offers an economy, mild condition, viable and scalable strategy for preparing highly efficient HER electrocatalysts for the development of effective electrochemical water-splitting technology.

## Additional file

**Additional file 1: Figure S1.** XRD spectra for MoS_x_ film grown on the Ti ingot by chronopotentiometry negative electrodeposition at 2 mA cm^−2^. **Figure S2.** Nyquist plot representations of electrochemical impedance spectra of S-0.4, S-0.4-SDS, and Ti ingot. **Figure S3.** SEM images and EDS elemental mapping for Mo and S of amorphous MoSx films. Panels a and d are the SEM images for S-0.4 (a) before and (b) after CV for 1000 cycles with corresponding (b, c, e, f) EDS elemental mapping images, respectively. Panels g and j are the SEM images for S-0.4-SDS (c) before and (d) after CV for 1000 cycles with corresponding (h, i, k, l) EDS elemental mapping images, respectively.

## Data Availability

We have presented all our main data in the form of tables and figures.

## Abbreviations

MoS_x_: amorphous molybdenum sulfide
HER: hydrogen evolution reaction
CP: chronopotentiometry
CV: cyclic voltammograms
LSV: linear sweep voltammetry
SCE: saturated calomel electrode
RHE: reversible hydrogen potential
EIS: electrochemical impedance spectroscopy
XRD: X-ray diffractometer
SEM: scanning electron microscopy
EDS: energy dispersive X-ray spectroscopic
CHI: electrochemical workstation
XPS: X-ray photoelectron spectroscopy
SDS: sodium dodecyl sulfate
PTFE: polytetrafluoroethylene
