# Supplementary material for: Electrodeposition of amorphous molybdenum sulfide thin film for electrochemical hydrogen evolution reaction
Source: BMC Chem. 2019 Jul 10;13(1):88. doi: 10.1186/s13065-019-0600-0 (PMC6661953; doi:10.1186/s13065-019-0600-0)
Supplement: Supplementary file 1 — Additional file 1: Figure S1. XRD spectra for MoSx film grown on the Ti ingot by chronopotentiometry negative electrodeposition at 2 mA cm−2. Figure S2. Nyquist plot representations of electrochemical impedance spectra of S-0.4, S-0.4-SDS, and Ti ingot. Figure S3. SEM images and EDS elemental mapping for Mo and S of amorphous MoSx films. Panels a and d are the SEM images for S-0.4 (a) before and (b) after CV for 1000 cycles with corresponding (b, c, e, f) EDS elemental mapping images, respectively. Panels g and j are the SEM images for S-0.4-SDS (c) before and (d) after CV for 1000 cycles with corresponding (h, i, k, l) EDS elemental mapping images, respectively. [file 13065_2019_600_MOESM1_ESM.docx]

Electrodeposition of Amorphous Molybdenum Sulfide Thin Film for Electrochemical Hydrogen Evolution Reaction

Lina Zhang, Liangliu Wu, Jing Li, Jinglei Lei^*^

Lina Zhang，*School of Chemistry and Chemical Engineering, Chongqing University, Chongqing 400044, People’s Republic of China. E-mail: lina4062@163.com.*

Liangliu Wu, *School of Chemistry and Chemical Engineering, Chongqing University, Chongqing 400044, People’s Republic of China. E-mail:* *980689062@qq.com.*

Jing Li, *School of Chemistry and Chemical Engineering, Chongqing University, Chongqing 400044, People’s Republic of China. E-mail: 13628357624@139.com.*

Jinglei Lei, *School of Chemistry and Chemical Engineering, Chongqing University, Chongqing 400044, People’s Republic of China. E-mail: leijlei@163.com*





Figure S1. XRD spectra for MoS_x_ film grown on the Ti ingot by chronopotentiometry negative electrodeposition at 2 mA·cm^-2^.





Figure S2. Nyquist plot representations of electrochemical impedance spectra of S-0.4, S-0.4-SDS, and Ti ingot.


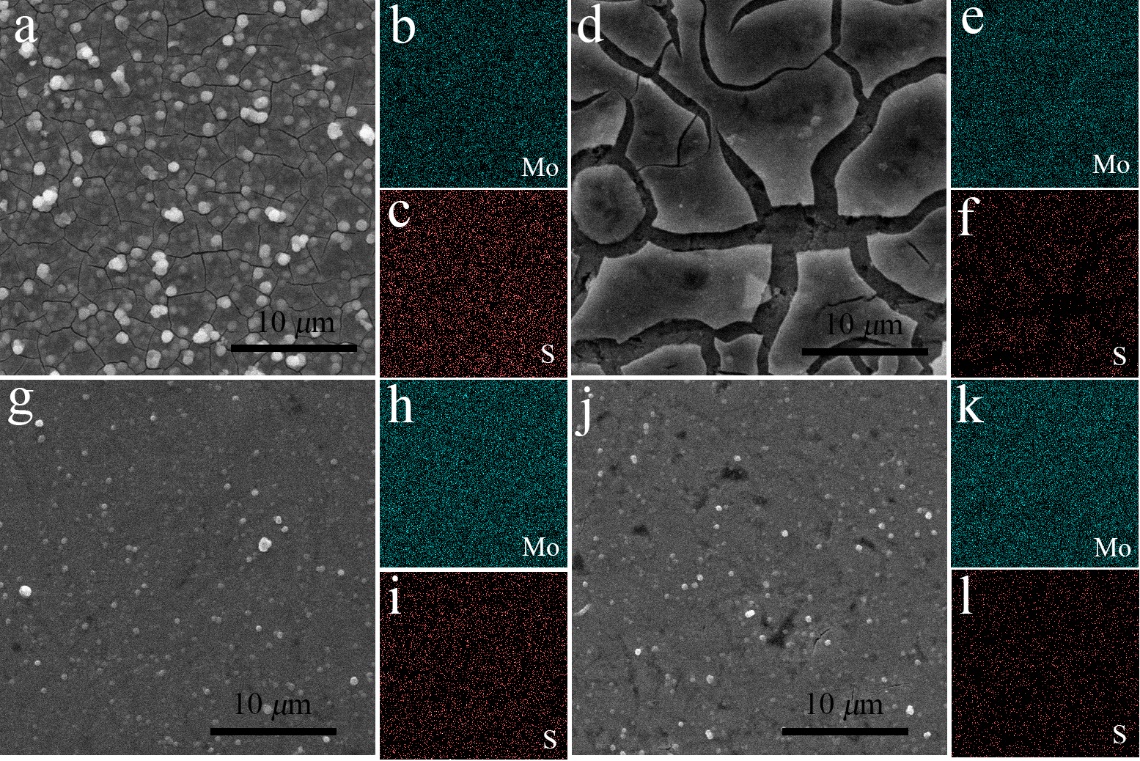


Figure S3. SEM images and EDS elemental mapping for Mo and S of amorphous MoSx films. Panels a and d are the SEM images for S-0.4 (a) before and (b) after CV for 1000 cycles with corresponding (b, c, e, f) EDS elemental mapping images, respectively. Panels g and j are the SEM images for S-0.4-SDS (c) before and (d) after CV for 1000 cycles with corresponding (h, i, k, l) EDS elemental mapping images, respectively.
